# The Impact of COVID-19 on Ophthalmology Practice: Changes and Controversies in Endophthalmitis Risk

**DOI:** 10.18502/jovr.v18i3.13771

**Published:** 2023-07-28

**Authors:** Mohammad Riazi

**Affiliations:** Department of Ophthalmology, Gavin Herbert Eye Institute, University of California Irvine, Irvine, California, USA

The COVID-19 pandemic imposed major risks to both ophthalmologists and patients, resulting in significant changes to ophthalmology practices around the world. Non-urgent ophthalmic services were temporarily halted, with a gradual reopening in a stepwise manner. In a 2020 survey conducted by the American Society of Retina Specialists (ASRS), about 40% of respondents indicated a reduction in staff. In some areas, ophthalmologists were redirected to serve in COVID-related areas such as emergency wards or intensive care units. The pandemic has also accelerated the adoption of virtual visits and digital patient management.

Detection of viral RNA particles in ocular secretions suggested the possibility of transmission through the eyes, resulting in recommendations for eye protection for ophthalmologists (such as slit lamp shields, goggles, or eye shields) and mandatory mask-wearing and temperature screenings for both patients and staff. Virtual visits were introduced in some areas to compensate for decreased patient numbers, and vaccination and testing protocols were established. Mask-wearing might be thought to reduce the rate of endophthalmitis by reducing exposure to nasopharyngeal and oral flora, but it may also increase the flow of air and germs from the nose and oral cavity to the eyes. Tight-fitting face masks used without adhesive tapes resulted in the highest amount of bacterial growth around the subject's eye.^[1]^ Sakamoto et al reported an increased rate of post-vitrectomy endophthalmitis in the COVID era, with more germs originating from the oral cavity.^[2]^

Several case reports have shown a link between COVID-19 and ocular disorders, including conjunctivitis, keratitis, uveitis, and retinal vascular occlusions. The pathogenesis, in some cases, involves attachment of viral spike proteins to cell receptors such as angiotensin-converting enzyme 2 (ACE-2) and cleavage by protein trans-membrane serine protease 2 (TMPRSS2) for cell entry in ocular tissues.^[3]^ However, other mechanisms of viral infection such as alterations in coagulation cascades or immune function may also play a role.

Presentation of various ophthalmic disorders has been reported to change during the COVID era. Most of these differences may be attributed to limited access to providers, use of personal protective devices, and patient hesitancy/self-isolation, causing delayed presentation or referral. During the lockdown period, Das and Dave reported an increase in proliferative retinopathy or sight-threatening conditions among diabetic patients.^[4]^ Reports have also suggested an association between COVID infection and fungal sepsis or fungal ophthalmic involvement.^[5]^

In the current issue of *Journal of Ophthalmic and Vision Research (JOVR)*, two papers address changes in the rate of endophthalmitis among tertiary ophthalmic centers during the COVID-19 pandemic. One paper by Dr. Karimi and colleagues^[6]^ examines the risk of acute endophthalmitis after intravitreal injections associated with the widespread use of face masks. These authors compared 28,085 injections in the pre-COVID era with 10,717 injections during the COVID era and found no significant differences in the rate of endophthalmitis after injections (0.032% in pre-COVID era vs 0.037% in COVID era). Although the use of masks helps limit the spread of the virus, it entails potential side effects, such as dry eye, infectious keratitis, or even endophthalmitis. In a study involving simulated intravitreal injections, Patel et al showed that adhesive taping at the superior edge of the patients' mask could decrease the rate of bacterial dispersion to the level of an N95 mask.^[1]^ In Karimi's paper patients wore masks during the COVID era, but held their masks under their nose during the injection, which avoids re-direction of air flow towards the eyes.

Another paper by Dr. Fortes and colleagues^[7]^ is a multi-center retrospective case series that evaluates clinical characteristics and visual outcomes of patients with endophthalmitis before and during the COVID-19 pandemic. The authors found similar etiology for endophthalmitis in both groups, as well as similar final visual outcomes at six months. However, the mean logMAR visual acuity at presentation was worse during the pandemic as compared to the pre-COVID period, which may be due to delayed presentation (18 days vs 7 days). Despite reports of irreversible damage in neovascular AMD, retinal vein occlusion, or retinal detachment due to delayed referral in the COVID era,^[8,9]^ Fortes^[7]^ did not find a significant difference in the final outcome in patients with endophthalmitis. On the other hand, Das and Dave^[4]^ reported an increase in the number of endogenous endophthalmitis and a decrease in post-traumatic forms during the COVID era, which is in contrast to the report by Fortes et al.^[7]^ Nevertheless, Fortes did not find a significant difference in the rate of post-injection endophthalmitis during the pandemic, which is in agreement with Karimi's paper and the IRIS registry study.^[10]^

In summary, the COVID-19 era has resulted in significant changes in ophthalmology practices worldwide, including delayed patient referrals, universal mask-wearing by both patients and providers, and direct/indirect ocular involvement by the virus. Although there are conflicting reports in the literature regarding the impact of these changes on the incidence of postoperative and post-injection endophthalmitis, the two studies included in this issue of *JOVR* provide evidence that there have been no significant changes in the rate of endophthalmitis during the COVID era.

##  Financial Support and Sponsorship

None.

##  Conflicts of Interest

None.
